# NADPH Oxidase-2 Derived ROS Dictates Murine DC Cytokine-Mediated Cell Fate Decisions during CD4 T Helper-Cell Commitment

**DOI:** 10.1371/journal.pone.0028198

**Published:** 2011-12-01

**Authors:** Meghan A. Jendrysik, Sam Vasilevsky, Liang Yi, Adam Wood, Nannan Zhu, Yongge Zhao, Sherry M. Koontz, Sharon H. Jackson

**Affiliations:** 1 Molecular Defenses Section, Laboratory of Host Defenses, National Institute of Allergy and Infectious Diseases, National Institutes of Health, Bethesda, Maryland, United States of America; 2 Monocyte Trafficking Unit, Laboratory of Host Defenses, National Institute of Allergy and Infectious Diseases, National Institutes of Health, Bethesda, Maryland, United States of America; 3 Clinical Immunology Unit, Laboratory of Host Defenses, National Institute of Allergy and Infectious Diseases, National Institutes of Health, Bethesda, Maryland, United States of America; University of Muenster, Germany

## Abstract

NADPH oxidase-2 (Nox2)/gp91^phox^ and p47^phox^ deficient mice are prone to hyper-inflammatory responses suggesting a paradoxical role for Nox2-derived reactive oxygen species (ROS) as anti-inflammatory mediators. The molecular basis for this mode of control remains unclear. Here we demonstrate that IFNγ/LPS matured p47^phox−/−^-ROS deficient mouse dendritic cells (DC) secrete more IL-12p70 than similarly treated wild type DC, and in an *in vitro* co-culture model IFNγ/LPS matured p47^phox−/−^ DC bias more ovalbumin-specific CD4^+^ T lymphocytes toward a Th1 phenotype than wild type (WT) DC through a ROS-dependent mechanism linking IL-12p70 expression to regulation of p38-MAPK activation. The Nox2-dependent ROS production in DC negatively regulates proinflammatory IL-12 expression in DC by constraining p38-MAPK activity. Increasing endogenous H_2_O_2_ attenuates p38-MAPK activity in IFNγ/LPS stimulated WT and p47^phox−/−^ DC, which suggests that endogenous Nox 2-derived ROS functions as a secondary messenger in the activated p38-MAPK signaling pathway during IL-12 expression. These findings indicate that ROS, generated endogenously by innate and adaptive immune cells, can function as important secondary messengers that can regulate cytokine production and immune cell cross-talk to control during the inflammatory response.

## Introduction

Mature dendritic cells (DC) are pivotal for T cell activation and polarization. DC utilize germline-encoded toll-like receptors (TLR) to recognize evolutionary conserved moieties on pathogens called pathogen-associated molecular patterns (PAMPs), thereby helping to ensure the immune response is appropriate for a particular pathogen [1], [2]. Following initial contact with a pathogen, DC migrate to the lymph nodes (LN) and spleen to stimulate naïve T cells [3]. Maturing DC increase cell surface major histocompatibility and co-stimulatory molecule expression, and secrete certain cytokines, notably IL-12 and IL-6, that are crucial to immunologic synapse formation, T cell activation and the polarization of T cells towards distinct Th phenotypes [4], [5]. Additionally, the nature of the pathogen determines which cytokines DCs secrete [3], [4], [6].

LPS derived from pathogens such as *Escherichia coli* is a well-characterized pathogen-associated molecular pattern that initiates a cascade of cytoplasmic signal transduction events downstream of TLR4, which include p38-MAPK modulation of IL-12p70 production and PI3K-mediated negative regulation of IL-12 transcription and cytokine secretion in activated DC [7], [8], [9], [10]. Investigations have also shown enhanced protein kinase C (PCK) α/β and ε phosphorylation in LPS stimulated DC, and that PKCε can regulate IL-12p70 secretion in monocyte-derived DC [11]. Although the full signal transduction pathway between LPS-TLR4 and IL-12p70 production remains undefined, evidence suggest that different signaling molecules cooperate to constrain IL-12 production and the inflammatory pathway. It has been shown that TLR signaling activates lipid products of PI3K including phosphatidylinositol-3,4-bisphosphate that recruit and activate protein kinase B (PKB)/AKT downstream of PI3K [12]. Thereby, PI3K activation leads to the eventual suppression IL-12p70 by recruiting and activating AKT-1, which phosphorylates glycogen synthase kinase 3 (GSK-3) [13], [14]. GSK-3 positively regulates DC IL-12p70 expression in DC [12], [14], [15] and its phosphorylation downstream of PI3K and AKT is inhibitory. Moreover, several other reports have shown that LPS induced PI3K activation also leads to the eventual suppression of IL-12p70 production in DC through inhibition of p38-MAPK activity [9], [14], although the molecular mechanism has not been delineated,

Coincident with their putative roles in IL-12p70 regulation, PKC and PI3K have both been shown to activate p47^phox^ via phosphorylation [16]. p47^phox^ is an adaptor protein that supports the multi-component membrane-associated, Nox2 enzyme that produces the well characterized phagocyte respiratory burst [17]. Mutations in the genes for p47^phox^ and the membrane-bound catalytic component Nox2/gp91^phox^ account for the majority of Chronic Granulomatous Disease (CGD) cases [18], [19]. Mice deficient in either one of these components develop CGD-like symptoms [20], [21], characterized by systemic infection, granulomatous inflammation and autoimmune disease. T cell blasts from mice deficient in Nox2 and p47^phox^ produced less T cell receptor (TCR) induced hydrogen peroxide (H_2_O_2_) and significantly more IFNγ, IL-2 and TNF-α than wild type (WT) T cell blasts when stimulated via the TCR complex [22]. This suggests that endogenously generated reactive oxygen species (ROS) may act in a manner similar to secondary messengers to dynamically influence cytokine expression. To determine the role of Nox2-dependent ROS activity in the regulation of DC cytokine secretion and consequent Th cell polarization we performed a series of *in vitro* experiments, which mimic *in vivo* DC-T cell interactions to assess the role of ROS in the modulation of cytokine expression during DC-T cell interactions. We show that similar to the role of GSK-3 inhibition downstream of PI3K and AKT; endogenous Nox2-derived ROS plays a major role in constraining IL-12p70 production in DC. In the absence of Nox2-dependent ROS, unrestrained IL-12p70 production drives CD4^+^ T cell fate decision toward Th1 differentiation. We show that a major target of Nox2-derived ROS is the p38-MAPK pathway. Our results show that Nox2 derived hydrogen peroxide (H_2_O_2_) acts in parallel with regulation by PI3K/AKT to control IL-12p70 production by negatively regulating IL-12p70 expression through p38-MAPK repression in murine DCs. These findings define a novel role for p38-MAPK and Nox2-dependent ROS in the homeostatic control of the inflammatory response through the dynamic mediation DC-T cell communication.

## Results

### p47^phox−/−^- ROS deficient DC show enhanced IL-12p70 secretion

**Figure 1:**
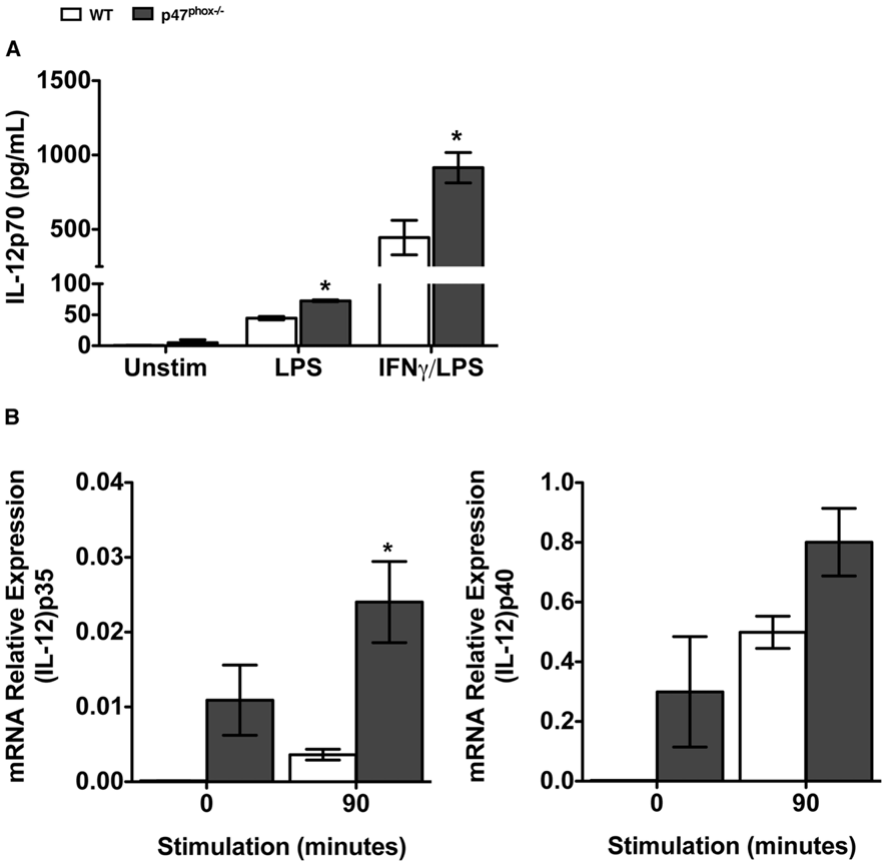
Enhanced IL-12p70 secretion by ROS deficient p47^phox−/−^ DC. One million immature DC were cultured in media alone (0) or primed with IFNγ for 3 hours then stimulated with LPS for 90 minutes as indicated. (A) Secreted IL-12p70 detected in supernatants of overnight cultures of immature, LPS and IFNγ/LPS stimulated DC. The data are the mean (± SEM) percentage for 5 individual experiments with 3–4 of each genotype/experiment. *p≤0.01, n = 5. (B) Immature DC were cultured in media alone or primed with IFNγ for 3 hours then stimulated with LPS 90 minutes as indicated. Following RNA extraction, IL-12p35 and IL-12p40 expression was quantitated by real-time PCR. The data are the mean (± SEM) percentage for 3 individual experiments with 3–4 of each genotype/experiment. *p<0.02.

IL-12p70 is the prototypic cytokine of a small family of heterodimeric cytokines, which also include Il-23, IL-27 and IL-35 [24], [25]. The IL-12- related cytokines contain an α chain (p19, p28 or p35) and a β chain (p40 or Ebi3). Both p40 and p35 need to be expressed in the same cell to produce the bioactive IL-12p70 protein [24], [26]. Recent investigations indicate that reactive oxygen species (ROS) modulate IL-12 expression in DC [27]. However, the source and location of ROS in DC, as well as the underlying mechanism(s) of ROS-dependent regulation of molecular signaling pathways for IL-12 expression are not yet defined. Given that Th1 cytokine expression is enhanced in TCR stimulated T cell blasts from both gp91^phox^/Nox2 catalytic subunit and p47^phox^ deficient mice we speculated whether ROS deficient DC could also be induced to secrete increased amounts of IL-12, a critical cytokine for the induction of IFNγ secretion in T cells and for the eventual skewing towards a Th1 cell fate decisions and responses [4]. As shown in Figure 1, we found that the amount of secreted IL-12p70 was higher in supernatants from both unstimulated and LPS stimulated p47^phox−/−^ DC, compared to WT DC. Several studies have shown that LPS induced IL-12p70 production is enhanced in the presence of IFNγ [24], [28]. Therefore in order to examine whether LPS activation of DC synergizes with IFNγ to make more IL-12, we primed immature DC with IFNγ for 3 hours prior to overnight LPS stimulation. We found that IFNγ primed WT and p47^phox−/−^ DC secreted nearly 20 times more IL-12p70 than DC stimulated with LPS alone, and that p47^phox−/−^ DC secreted nearly twice as much IL-12p70 as WT DC (Figure 1A).

Out of the panel of cytokines analyzed, IFNγ/LPS stimulation triggered enhanced IL-12p70 and IL-6 expression in p47^phox−/−^ DC compared to similarly treated WT DC. Of note is that there was no difference in the amount of constitutively or IFNγ/LPS stimulated IL-12/IL-23p40 (Table 1), which suggest that the enhanced IL-12p70 secretion by p47^phox−/−^ DC may be due to aberrant regulation of the IL-12p35 subunit [24]. IL-12p70 production is primarily regulated at the level of transcription [8], [29]. Therefore, we used quantitative real-time PCR to establish whether the enhanced IL-12p70 production by p47^phox−/−^-ROS deficient DC is regulated at the level of transcription. As shown in Figure 1B, the transcription of IL-12p35 was significantly enhanced in IFNγ/LPS stimulated p47^phox−/−^ DC compared to WT DC. Collectively these findings implicate a role for Nox2-dependent ROS in modulating IFNγ -mediated enhanced IL12p35 transcription [24] and IL-12p70 heterodimer secretion in DC.

**Table 1:** IFNγ/LPS induced DC cytokine secretion.

	Unstimulated		LPS+IFNγ	
	WT	p47^phox−/−^	WT	p47^phox−/−^
IL-12p70	10.67 (0.984)	21.62 (2.354)*	206.7 (14.10)	396.9 (31.93)**
IL-12p40	69.66 (3.84)	52.33 (6.88)	5654 (95.81)	5692 (89.91)
IL-23	92.5 (12.8)	99.4 (13.9)	131.6 (9.3)	151.2 (9.7)
IL-6	6184 (1586)	9867 (790.4)	15050 (2694)	29100 (2484)*

Mean (SEM) in pg/mL,

*p≤0.003,

**p = 0.0006, n = 5 IL-12p70 and IL-6. n = 3 IL-12p40 and IL-23.

DC were generated as described, and matured overnight with IFNγ (200 ng/mL) and LPS∶B8 (0.1 µg/mL). Supernatants were assayed by ELISA.

### p47^phox−/−^ DC induce enhanced IFNγ expression in OT-II lymphocytes

Since cytokine expression is enhanced in both ROS deficient p47^phox−/−^ DC and T cells, we hypothesized that the positive feed-forward signal amplification between these cell types is dynamically regulated by Nox2-dependent ROS to constrain T cell-derived IFNγ stimulation of DC IL-12p70 secretion during Th1 inflammatory responses. To assess this, we examined WT and p47^phox−/−^ DC induced T cell activation, cytokine expression, and polarity using ovalbumin-specific transgenic (OT-II) CD4^+^ lymphocytes. For these investigations we matured bone marrow derived DC from p47^phox−/−^ and WT mice overnight with IFNγ and LPS, and pulsed them with the OVA_323–339_ peptide, as described in the methods.

Co-cultures of OVA_323–339_ peptide-pulsed mature DC and OVA-specific CD4^+^ (OT-II) T lymphocytes showed no differences, between WT and p47^phox−/−^ DC stimulated OT-II cells, in the percentage of cells activated (as determined by the forward scatter during FACS analysis), viability, or the expression of activation markers CD25 and CD69. There was likewise no difference in the CFSE profiles, indicating similar rates of proliferation (Figure S1).

**Figure 2:**
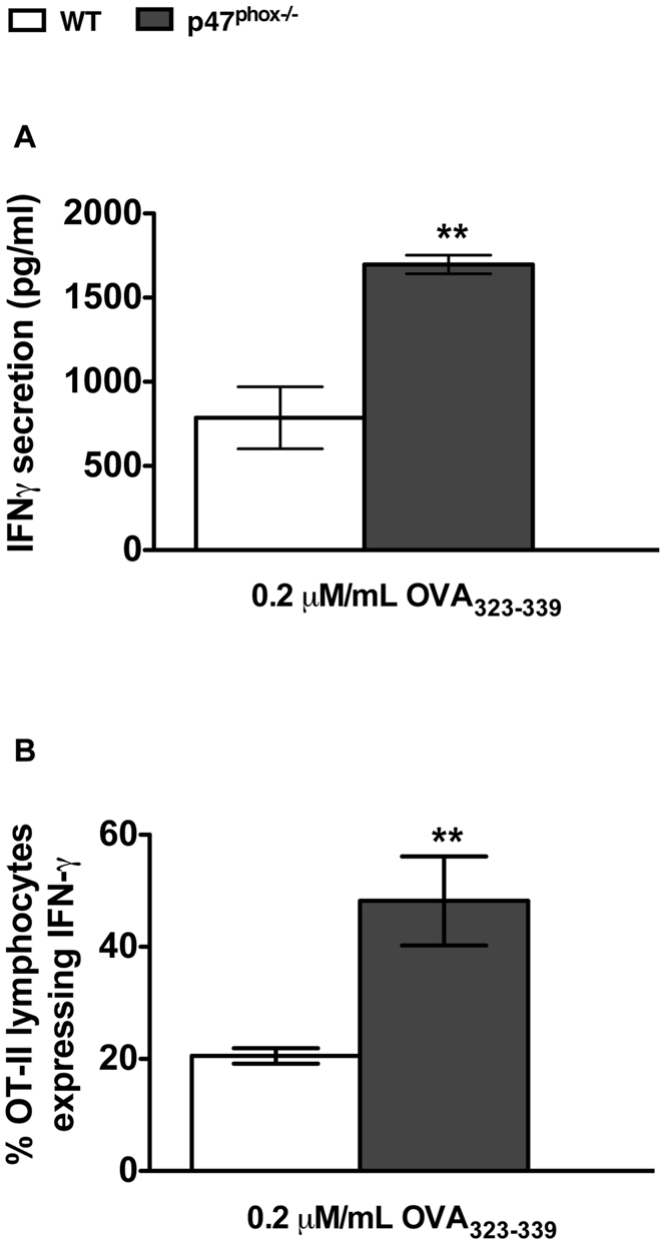
p47^phox−/−^ DC bias more OT-II T lymphocytes to secrete IFNγ. OT-II lymphocytes were stimulated with mature OVA_323–339_ peptide-pulsed DC for 96 hours. (A) Secreted IFNγ detected in supernatants of 96 hour DC-OT-II co-cultures. The stimulated OT-II lymphocytes were rested in medium supplemented with rIL-2 for six days, and then restimulated with PMA and ionomycin for 4 hours, with monensin added for the last 2 hours. (B) Intracellular IFNγ production was then assessed by flow cytometry. Percentages of OT-II lymphocytes expressing IFNγ. The data are the mean (± SEM) percentage for 4 individual experiments with 3–4 of each genotype/experiment ** *p*<0.002.

The level of IFNγ measured in the supernatants of p47^phox−/−^ DC–OT-II co-cultures was 2-fold higher with 0.2 µM OVA_323–339_ peptide-pulsed p47^phox−/−^ DC compared to similarly treated WT DC (Figure 2A). Additionally, the levels of IL-2, IL-6 and IL-17 were not increased (Table 2), which strongly suggest that this is a phenomenon specific to IFNγ production. To confirm that the OT-II lymphocytes were biased during the DC-T cell co-culture, the stimulated OT-II cells were rested for 6 days in IL-2 supplemented medium, and then restimulated with PMA and ionomycin for 4 hours in the presence of monensin for the final two hours. Again, OT-II cells stimulated by p47^phox−/−^ DC that were loaded with 0.2 µM/mL OVA_323–339_ peptide showed a significant increase (2.3-fold) in the percentage of viable cells expressing IFNγ compared to those stimulated by WT DC (Figure 2B). These results indicate a dynamic cross-talk between DC and T cells that drives the Th1 inflammation response through T cell derived IFNγ modulation of DC IL-12p70 production.

**Table 2:** Primary DC-OT-II lymphocyte co-culture cytokine secretion.

[OVA_323–339_]	0.2 µM		0	
	WT	p47^phox−/−^	WT	p47^phox−/−^
IL-2	1688(499.7)	1127(400.1)	385.4(236.7)	51.80(51.8)
IL-4	386.8 (260.8)	221.7 (160.7)	49.71(49.71)	0(0)
IL-6	2386(745.7)	2409(598.2)	1140(353.7)	941(139.5)
IL-17	312.4(59.62)	559.0(131.2)	275.3(75.23)	49.98(49.98)

Mean (SEM) in pg/mL, n = 4.

IFNγ/LPS matured DC were pulsed for 2 hours with OVA_323–339_ peptide at the indicated concentrations, and cocultured with naïve OT-II CD4^+^ lymphocytes. Supernatants were collected at 96 hours, and analyzed by a bead-based multiplex ELISA.

### p47^phox^ attenuates IL-12p70 expression by integrating PI3K-dependent negative regulation of p38-MAPK

**Figure 3:**
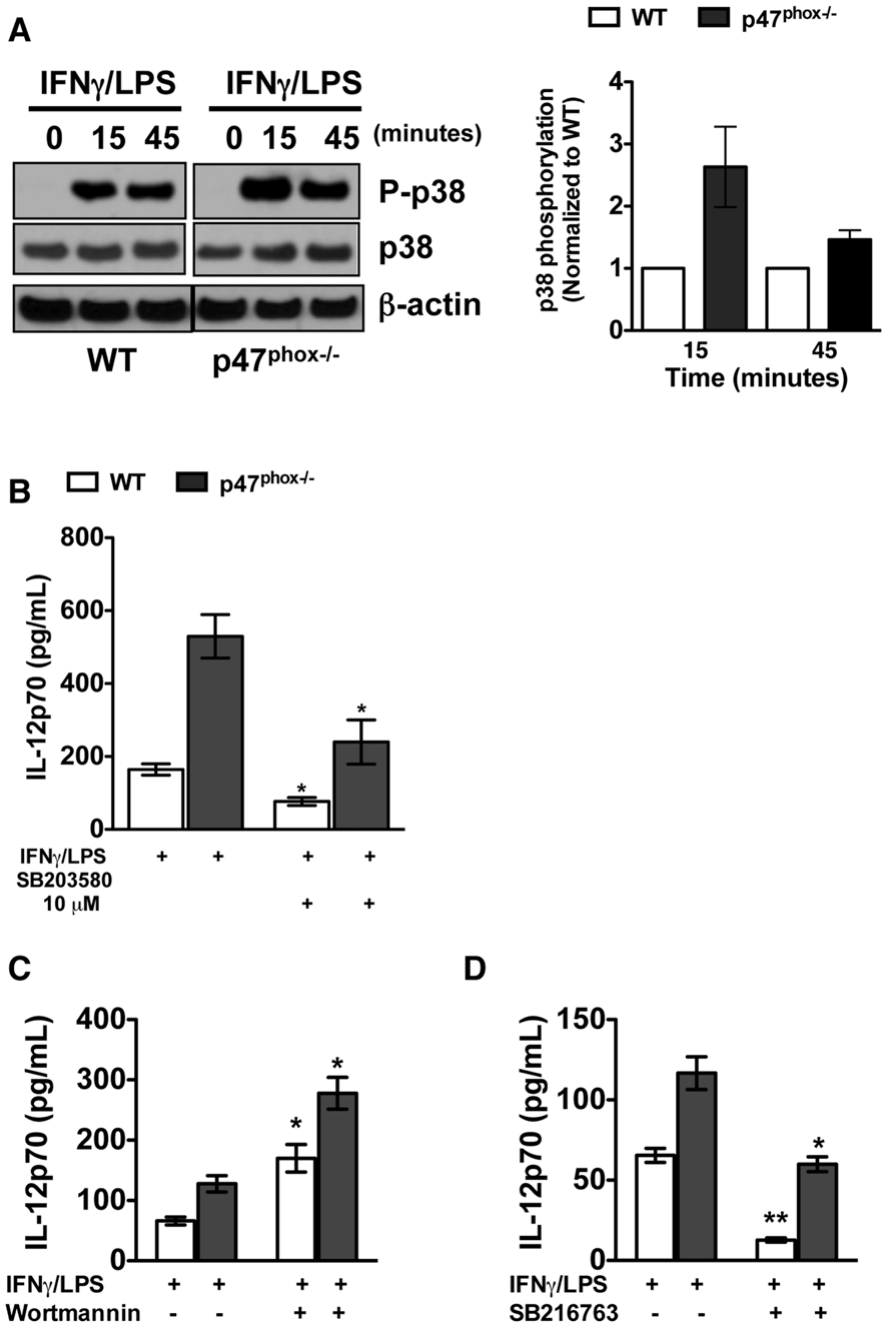
p47^phox^ attenuates IL-12p70 expression through PI3K-dependent negative regulation of p38-MAPK. (A) Detection of phosphorylated and total p38-MAPK was determined by Western blot of immature DC, 1×10^6^/ml, stimulated with IFNγ/LPS for 0, 15, and 45 minutes as indicated (Density plot for n = 4 experiments with 3 of each genotype/experiment). (B) Secreted IL-12p70 in supernatants from IFNγ/LPS stimulated DC treated, or not, with SB203580. The data are the mean (± SEM) percentage for 5 individual experiments with 3–4 of each genotype/experiment, with 5×10^5^ DC/0.5 ml. Supernatants from IFNγ/LPS-stimulated DC treated, or not, with Wortmannin (C) or SB216763 (D) were harvested from overnight cultures and assayed for secreted IL-12p70. The data are the mean (± SEM) percentage for 3 individual experiments with 3–4 of each genotype/experiment with 2×10^5^ DC/0.2 ml. * p<0.003, **p≤0.0003.

In additional investigations we found that IFNγ/LPS stimulation induced comparable upregulation of surface costimulatory markers CD40, CD80, CD86, surface MHC-II, and toll like receptor (TLR)-2 and TLR-4 on both WT and p47^phox−/−^ DC (data not shown). Finding that IL-12p70 secretion is enhanced in the mature p47^phox−/−^ DC, and that there is no difference in the immuno-phenotypic maturation of WT and p47^phox−/−^ DC following IFNγ/LPS stimulation suggest that the increased IL-12p70 production in p47^phox−/−^ DC is a result of aberrant signal transduction downstream of LPS/IFNγ. p38-MAPK is a critical regulator of IL-12 transcription in macrophages and DC [8], [29], and it is has been shown that nonspecific inhibition of the PI3K pathway with wortmannin enhances LPS-induced p38-MAPK phosphorylation [8], [13]. We used Western analysis to examine the role of p38-MAPK in regulating IFNγ/LPS stimulated IL-12p70 expression in p47^phox−/−^ DC. Total p38-MAPK was constant at all time points for WT and p47^phox−/−^ DC. However, p38-MAPK was hyper-phosphorylated in IFNγ/LPS stimulated p47^phox−/−^ DC relative to WT DC (Figure 3A). We also found that 10 µM of p38-MAPK specific inhibitor SB203580 significantly suppressed IFNγ/LPS stimulated IL-12p70 secretion in both WT and p47^phox−/−^ DC treated (Figure 3B).

To investigate the influence of other signal transduction kinases in the regulation of IL-12p70 in p47^phox−/−^ DC, we determined IL-12p70 secretion by p47^phox−/−^ DC treated with kinase inhibitors. Although an inhibitor peptide for PKCε was shown to positively regulate LPS induced IL-12 secretion in human DC [11], we found that the PKCε inhibitory peptide did not affect WT or p47^phox−/−^ DC IL-12p70 secretion (Figure S2). We also found that the pan-PKC inhibitor bisindolylmaleimide inhibited IFNγ/LPS induced IL-12p70 secretion similarly in both p47^phox−/−^ and WT DC. Hence, these investigations indicate that the enhanced p47^phox−/−^ DC IL-12p70 secretion is not mediated by PKC.

We also found that non-specific inhibition of the PI3K pathway with wortmannin similarly enhanced IL-12p70 production by both WT and p47^phox−/−^ DC during overnight stimulation (Figure 3C), suggesting that the enhanced IL-12p70 secretion by wortmannin treated p47^phox−/−^ DC was due to the suppression of a negative regulator of IL-12 production, and most probably downstream and or in parallel to PI3K. Next we examined downstream targeting by PI3K kinase activity to discern whether p47^phox^ may function as a negative or positive regulator in the PI3K pathway to control DC IL-12p70 production. As shown in Figure 3D the targeted inhibition downstream of AKT using the GSK-3 inhibitor SB216763 suppressed IL-12p70 production in WT and p47^phox−/−^ DC, with larger influence (2.5 fold) on WT DC compared to p47^phox−/−^ DC. Collectively, these investigations indicate that the enhanced secretion of IL-12p70 by p47^phox−/−^ DC is controlled by two parallel pathways downstream of PI3K, and regulated by AKT/GSK-3 and p47^phox^ respectively. Interestingly, the data also indicate that there is more residual IL-12p70 secreted by p47^phox−/−^ DC that were treated with the GSK-3 inhibitor than by p47^phox−/−^ DC that were treated with the p38-MAPK inhibitor, which suggest that ROS regulation of the p38-MAPK pathway is more dominant in IFNγ/LPS stimulated DC.

### Role of Nox2-derived reactive oxygen species in IFNγ/LPS induced DC IL-12p70 expression

**Figure 4:**
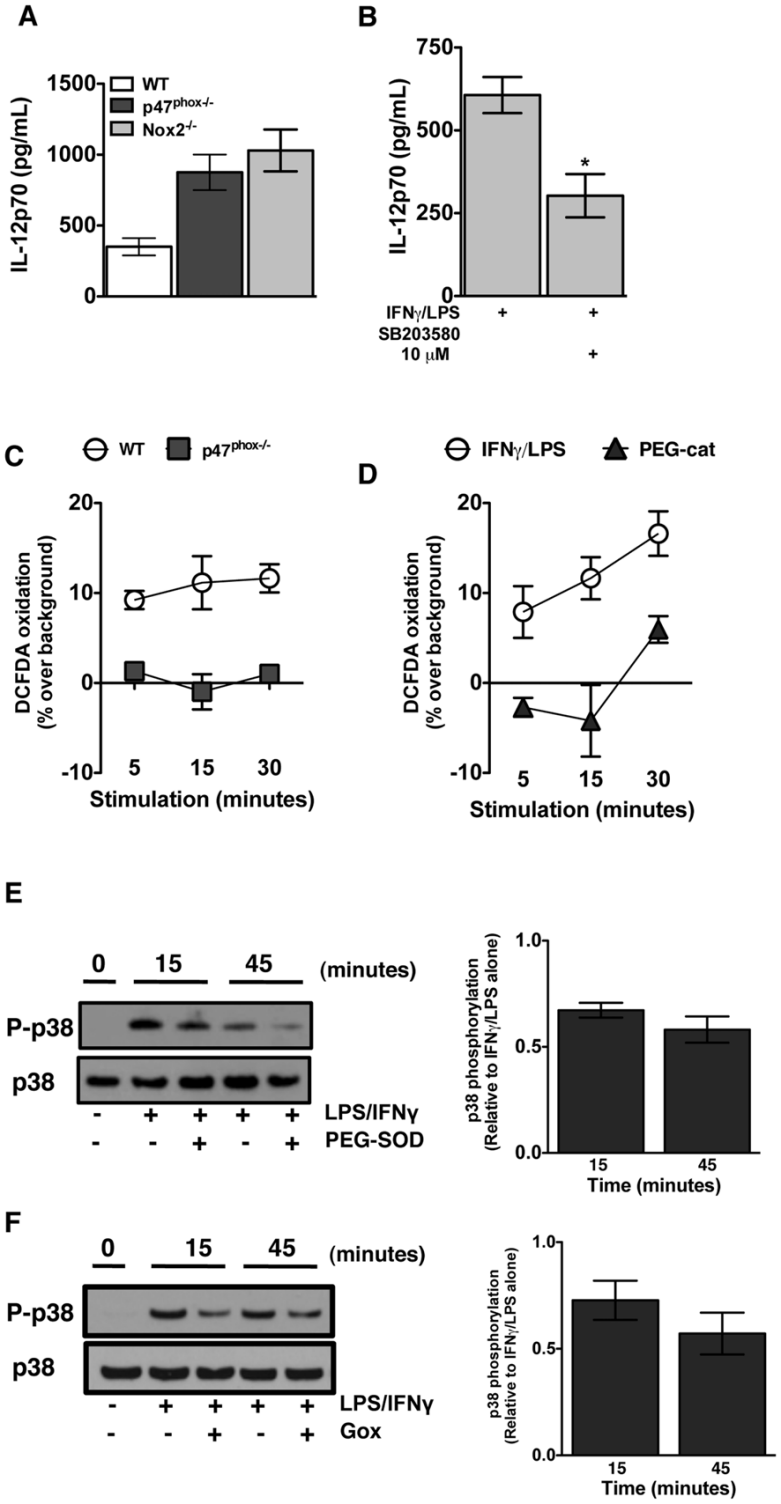
Nox2-derived H_2_O_2_ is a critical secondary messenger for IFNγ/LPS induced DC IL-12p70 expression. (A) Enhanced secretion of IL-12p70 detected in IFNγ/LPS stimulated DC supernatants from p47^phox^ and Nox2^−/−^ mice. (B) Enhanced secretion of IL-12p70 detected in IFNγ/LPS stimulated DC supernatants from Nox2^−/−^ mice treated, or not, with SB203580. The data are the mean (± SEM) percentage for 5 individual experiments with 3 of each genotype/experiment, with 0.5–1×10^6^ DC, respectively. Immature DC were stimulated with IFNγ/LPS for 0, 5, 15, and 30 minutes. All samples were pulsed with 2 µM DCFDA for the final 15 minutes of incubation. DCFDA dye oxidation in WT and p47^phox−/−^ DC stimulated with IFNγ/LPS alone (C) or in (D) WT DC pretreated with 200 units/ml PEG-catalase. The data are the mean (± SEM) percentage for 4 individual experiments with 3–4 of each genotype/experiment. Expression of total and phosphorylated p38-MAPK was determined by Western blot in (E) immature WT DC stimulated with IFNγ/LPS alone or with PEG-SOD (Density plot for n = 4 experiments with 3 of each genotype/experiment), and (F) immature p47^phox−/−^ DC stimulated with IFNγ/LPS alone or in with 0.01 µg/ml GOx (Density plot for n = 5 experiments with 3 of each genotype/experiment, as indicated.

These investigations implicate a role for Nox2-derived ROS in attenuating DC proinflammatory IL-12p70 production by integrating the PI3K-dependent negative regulation of p38-MAPK. However, an important question raised by these data is the relative roles of the p47^phox^ component of the Nox2 complex, and the ROS produced by the fully assembled and activated Nox2 holoenzyme. A recent report showed that p47^phox^, independent of Nox2, negatively regulates TLR-9 induced IL-12p70 expression in splenic DC [30]. To address this, we used ELISA to assess IFNγ/LPS stimulated IL-12p70 secretion in Nox2 catalytic subunit Nox2^−/−^ DC. As shown in Figure 4A, IL-12p70 production is also enhanced in IFNγ/LPS stimulated Nox2^−/−^ DC. Additionally, similar to p47^phox−/−^ DC, 10 µM of p38-MAPK inhibitor SB203580 also suppressed IFNγ/LPS stimulated IL-12p70 secretion in Nox2^−/−^ DC (Figure 4B). These findings indicate that active Nox2 enzyme-derived ROS, rather than the p47^phox^ protein alone, is required for IFNγ/LPS induced IL-12p70 regulation.

Elsen *et al.* suggested that DC express Nox2 component proteins and produce superoxide anion (O_2_^•−^) in a cryptic manner because of the presence of a membrane bound inhibitor that prevents Nox2 activation [31]. However, the inhibition was relieved with prolonged incubation with pro-inflammatory ligands such as LPS [31]. To further discriminate a role for intracellular Nox2-derived ROS in the PI3K-dependent negative regulation of IL-12 expression we used the substrate specific detection reagents DHE and DCFDA to assess early superoxide (O_2_^•−^) and hydrogen peroxide (H_2_O_2_) production, respectively, in IFNγ/LPS stimulated DC. IFNγ/LPS stimulation did not induce detectable DHE oxidation in WT or p47^phox−/−^ DC (data not shown). However, DCFDA was oxidized, 10% over background DCFDA oxidation, in WT DC stimulated with IFNγ/LPS (Figure 4C). Thus, although IFNγ/LPS stimulation did not induce early DHE-detectable O_2_^•−^ production it does induce low-level intracellular DCFDA-detectable H_2_O_2_ production in DC. Additionally, the importance of Nox2 enzymatic activity in IFNγ/LPS-induced ROS production was also demonstrated by the failure of IFNγ/LPS to induce DHE or DCFDA oxidation in p47^phox−/−^ DC (Figure 4C). In additional investigations we used PEG-catalase to scavenge IFNγ/LPS-induced H_2_O_2_ production detected by DCFDA oxidation in WT DC (Figure 4D), and thereby verified that the IFNγ/LPS-elicited ROS was H_2_O_2_.

To further assess the influence of intracellular Nox2-derived ROS on p38-MAPK phosphorylation, we stimulated WT DC with IFNγ/LPS in the presence or absence of the membrane-permeable ROS scavenger polyethylene glycol-superoxide dismutase (PEG-SOD), which converts O_2_^•−^ into H_2_O_2_. Interestingly, we found that PEG-SOD treated WT DC had reduced p38-MAPK phosphorylation (Figure 4E). Thus indicating that DC release early Nox2-derived O_2_^•−^ that is converted to H_2_O_2_ in response to IFNγ/LPS stimulation to repress p38-MAPK. To confirm this hypothesis we used glucose oxidase (GOx); a flavoenzyme that catalyses the conversion of β-D-glucose to H_2_O_2_ and produces a continuous pulse of intracellular H_2_O_2_ [32], [33], to restore H_2_O_2_ in IFNγ/LPS stimulated p47^phox−/−^ DC. As shown in Figure 4F, restoring intracellular H_2_O_2_ in IFNγ/LPS stimulated p47^phox−/−^ DC reduced phospho-p38-MAPK by 25% at 15 minutes and 43% at 45 minutes. Collectively these results indicate that Nox2-derived H_2_O_2_ negatively regulates p38-MAPK activation and thereby attenuates DC IL-12 secretion.

## Discussion

Pathogen stimulation induces DC to upregulate cell surface molecules and to secrete cytokines that determine T helper cell fate choice. Consequently, DC–T cell cross talk is critical to ensure that the appropriate inflammatory response is mounted to assure the immune response is appropriate for a particular pathogen. This study reports our finding that relative to WT DC, IFNγ/LPS stimulated Nox2-dependent ROS deficient DC secrete more IL-12p70 and thereby confirms a functional consequence of endogenous ROS production as a critical regulator of signaling pathways that control DC cytokine expression. Furthermore, our data demonstrates that Nox2-dependent ROS deficient DC bias more ovalbumin-specific OT-II lymphocytes to secrete IFNγ. The enhanced OT-II IFNγ production is independent of the number of OT-II cells stimulated and their level of stimulation as determined by the expression of the T cell activation markers CD69 and CD25, which indicates that the increased IFNγ production is not due to a difference in the robustness of the initial stimulation of the T cells. Rather, excess IL-12p70, secreted by DC deficient in endogenous ROS, initiates a dynamic communication between DC and T cells, and due to the relative potency of the DC-derived IL-12p70 the OT-II lymphocytes are driven to produce more IFNγ. The net effect is a self-amplifying process that leads to enhanced OT-II lymphocyte polarization and Th1 inflammation. Thus, although ROS can cause extensive tissue damage, our investigations demonstrate the critical role for endogenous ROS in regulating the production of inflammatory mediators that balance inflammation and control tissue homeostasis.

**Figure 5:**
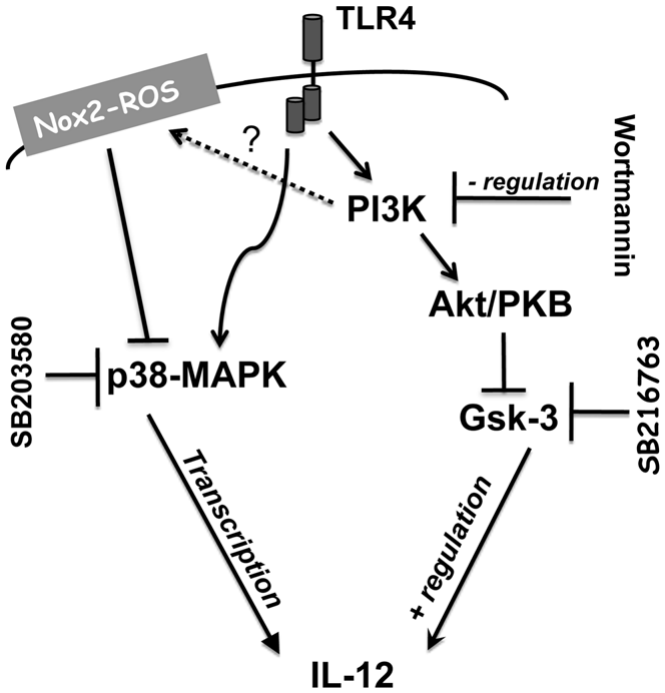
A schematic diagram of the positive and negative regulation of IL-12p70 expression. Nox2-dependent ROS regulation of p38-MAPK represses DC IL-12p70 production.

Recent reports demonstrate the significance of ROS in the regulation of IL-12p70 production [27], [34]. However, the endogenous source and subcellular localization of ROS were unclear. Vulcano *et al.* reported that Nox2-derived ROS did not influence mature human monocyte-derived DC IL-12 production [35]. However, it has been demonstrated that IL-12 is optimally induced in immature human monocyte-derived DC when the stimulus is given at the onset of maturation [36]. Thus, our finding that Nox2-derived ROS regulates IL-12p70 production in immature mouse DC that are matured with IFNγ and LPS, reveals new insight on the role of Nox2-derived ROS in the regulation of IL-12p70 protein production in immature versus mature DC. Additionally, it has been reported that DC propagated in GM-CSF generate more Nox-independent ROS as they differentiate from bone marrow hematopoietic progenitor cells into immature DC [27]. Our results, using p47^phox^ and catalytic subunit Nox2 deficient DC, indicate that Nox2-dependent ROS is necessary for regulating DC IL-12p70 expression. Our proposed model is consistent with microbial stimulation of Nox2-dependent O_2_^•−^ within phagosomal compartments, that is rapidly converted to more stable and diffusible H_2_O_2_, which is a decisive intermediary for parallel regulatory targets within PI3K and p38-MAPK signaling pathways that control IL-12p70 expression (Figure 5). Although the role of p38-MAPK in the regulation of IL-12p70 expression is not yet fully understood, our investigations reveal that Nox2-dependent H_2_O_2_ modulates p38-MAPK activation, and that there is enhanced IL-12p35 transcription in IFNγ/LPS stimulated Nox2-ROS deficient DC. Thereby, our data strongly suggests a role for endogenous Nox2-dependent ROS upstream of p38-MAPK.

By examining multiple signaling intermediates that have been implicated to both positively and negatively regulate IL-12 production we determined that multi-component Nox2-dependent regulation of IL-12p70 transcription is downstream of TLR-4 and PI3K. Activated PI3K lipid products such as phosphatidylinositol-3,4 bisphosphate (PtdIns(3,4)P_2_) have been shown to recruit and activate AKT [12], [37]. In the context of cytokine production, activated AKT phosphorylates specific targets such as Gsk-3, which regulate multiple transcription factors to balance pro- and anti-inflammatory cytokine expression [12], [13], [37]. Rodionova *et al.* showed that AKT inhibits the activity of GSK-3 via the phosphorylation of targeted serine residues, and consequently down-regulates IL-12 secretion [13]. We found that the GSK-3 inhibitor SB216763 only partially inhibited IL-12p70 production in p47^phox−/−^ DC, which indicates that this downstream parallel pathway maybe less dominant in p47^phox−/−^ DC.

Although the mechanism is unclear, it has been previously suggested that PI3K can mediate positive regulation of IL-12 via p38-MAPK, in macrophages and DC [8], [14], [38]. Our investigations showed that 10 µM of the p38-MAPK inhibitor SB203580 suppressed IL-12p70 secretion in WT and phagocyte oxidase deficient DC. In addition, we also found that higher dose (20 µM) SB203580 further inhibited IFNγ/LPS stimulated IL-12p70 secretion. However, this higher dose may also inhibit other PI3K target kinases, including AKT and GSK-3, involved in regulating IL-12p70 expression [39].

The importance of Nox2-dependent ROS for p38-MAPK-mediated IL-12p70 regulation was reinforced by finding that IFNγ/LPS-induced p38-MAPK phosphorylation is significantly enhanced in p47^phox−/−^ DC compared to similarly treated WT DC. Furthermore, while this regulation is more robust and dominant than AKT-mediated attenuation of IL-12 expression, collectively these investigations indicate that Nox2-derived H_2_O_2_ functions as a secondary messenger to integrate IFNγ/LPS activated PI3K, p38-MAPK and AKT signaling pathways during IL-12 expression.

Using substrate-specific detection reagents to distinguish O_2_^•−^ and H_2_O_2_, we observed that IFNγ/LPS induced low-level, catalase-inhibitable intracellular H_2_O_2_ in WT but not p47^phox−/−^ DC. Notably however, O_2_^•−^ production was not detectable following IFNγ/LPS stimulation in WT or p47^phox−/−^ DC using the substrate specific detector DHE. Nonetheless, we did find that pretreatment with PEG-SOD, to catalyze the dismutation of O_2_^•−^ into oxygen and H_2_O_2_, decreased the level of IFNγ/LPS stimulated p38-MAPK phosphorylation in WT DC. This indicates that IFNγ/LPS does trigger early O_2_^•−^ production in DC. Additionally, finding that in the presence of the O_2_^•−^ scavenger SOD, which converts O_2_^•−^ to H_2_O_2_, IFNγ/LPS stimulation reduced, not enhanced, p38-MAPK phosphorylation further supports that Nox2-derived H_2_O_2_ is a secondary intermediate that integrates p38-MAPK signaling in DC. To test this, we used glucose oxidase (GOx) [32], [33] to generate intracellular H_2_O_2_ in p47^phox−/−^ DC. Indeed, restoring intracellular H_2_O_2_ attenuated p38-MAPK hyper-phosphorylation in IFNγ/LPS stimulated p47^phox−/−^ DC. Thus, using substrate specific detection reagents and ROS scavengers we were able to discriminate that IFNγ/LPS induces early low-level intracellular O_2_^•−^ and H_2_O_2_ reactive oxygen intermediates, which forms the basis of our proposed model that multi-component Nox2-dependent ROS modulate IFNγ/LPS activated PI3K-MAPK signaling during IL-12 expression. In recognition of the constraints of these assays we also demonstrated that using GOx, rather than a H_2_O_2_ bolus to avoid the potential of causing oxidative stress in our *in vitro* culture model [40], [41], significantly dampens the observed IFNγ/LPS stimulated p38-MAPK phosphorylation in p47^phox−/−^ DC.

Our study demonstrates that low-level phagocyte oxidase-dependent ROS are regulatory, and that the loss of such deliberate regulatory ROS promotes inflammation. Accordingly, further investigations are required to determine whether modulation of ROS targets is a practical clinical therapeutic intervention for regulating inflammation. Given that in addition to infectious manifestations CGD patients are also prone to develop hyper-inflammatory and autoreactive diseases [42], [43], [44], IL-12p70 overexpression may have significant clinical relevance.

## Materials and Methods

### Ethics Statement

This study was carried out in strict accordance with the recommendations in the Guide for the Care and Use of Laboratory Animals of the National Institutes of Health/National Institute of Allergy and Infectious Diseases. This study, permit number ASP LHD 11, was reviewed and approved by the Animal Care and Use Committee of the National Institute of Allergy and Infectious Disease of the National Institutes of Health (Public Health Service Assurance A4149-01).

### Animals

Congenic p47^phox−/−^ mice [20] on a C57BL/6NTac background, WT C57BL/6NTac mice and C57BL/6-[Tg] TCR OT-II (OT-II) mice were obtained from Taconic Farms, Inc. (Hudson, NY). Nox2^−/−^ B6.129S6-*Cybb^tm1Din^*/J mice [21] were obtained from The Jackson Laboratory (Bar Harbor, ME).

### Reagents

Polyethylene glycol SOD (PEG-SOD), PEG-catalase, glucose oxidase (GOx), PMA and ionomycin were purchased from Sigma-Aldrich (St. Louis, MO). Wortmannin, bisindolylmaleimide and SB203580 were purchased from Calbiochem (EMD Chemicals, Inc., Gibbstown, NJ), SB216763 from Tocris (Ellisville, MO), and the PKCε inhibitory peptide from Santa Cruz Biotechnology, Inc. (Santa Cruz, CA). Inhibitors of signal transduction kinases associated with IL-12p70 regulation were titered to determine working concentrations that had a minimal effect on cellular viability. CFSE was purchased from (Molecular Probes/Invitrogen Corp, Carlsbad, CA).

### Isolation and culture of bone marrow derived dendritic cells

DC were harvested and cultured as described [22]. On day 6, the non-adherent cells have the immature DC phenotype CD11c^+^CD11b^+^MHCII^+^CD80^+^CD40^low^CD86^low^ [22]. Immature DC were primed with IFNγ (200 ng/1×10^6^ cells/mL R&D Systems Inc., Minneapolis, MN) for 2–4 hours, then matured overnight with LPS from *E. coli* (0.1 µg/1×10^6^ cells/mL 0127:B8, Sigma-Aldrich). As indicated 0.25–1×10^6^ immature DC where used. DC phenotype was determined by FACS by gating on CD11c^+^ (eBiosciences, San Diego, CA) cells using a FACSCanto (BD Pharmingen, San Diego, CA).

### DC – T lymphocyte co-cultures

Mature DC were pulsed with 0.2 µM/mL of OVA_323–339_ peptide (ISQAVHAAHAEINEAGR-17, NIAID Research Technologies Branch, Rockville, MD) for 2 hours, and then co-cultured at a 1∶1 ratio with CFSE-labeled splenic OVA-specific CD4^+^ (OT-II) T lymphocytes for 72–96 hours. Stimulated OT-II lymphocytes were rested in rIL-2 (100 U/mL, Rohmann-LaRoche Inc, Nutley, NJ) supplemented medium for 6 days and then restimulated with PMA (10 ng/ml) and ionomycin (1 µM) for 4 hours. Monensin (GolgiStop, BD Pharmingen) was added for the last 2 hours of stimulation. Cells were stained for CD4 and intracellular IFNγ (BD Pharmingen).

### Cytokine detection

Standard ELISA was performed for IL-12p70, IL-12p40, IL-6 and IFNγ (R&D Systems) and IL-23 (eBioscience) according to the manufacturers' instructions. Multiplex flow-based assays (FlowCytomix, Bender Medical Systems, Burlingame, CA) were performed according to manufacturer's instructions.

### Immunoblotting

For detection of phospho-p38-MAPK and p38-MAPK 10 µg clear cell lysate was resolved in SDS-PAGE and probed with anti-phospho-p38-MAPK and anti-p38-MAPK (Cell Signaling, Danvers, MA). For the relative quantification of the proteins scanned images were analyzed using ImageJ.

### Reactive oxygen species (ROS) assay

Intracellular ROS was assessed by dihydroethidium (DHE) or 2,7′ dichlorodihydrofluorescein diacetate (DCFDA) (Invitrogen, Carlsbad, CA) oxidation monitored by flow cytometric analysis as previously described [23]. For the PEG-catalse inhibition assay cells were washed twice with 1% BSA in PBS, then resuspended at 1×10^6^ cells/mL in 1% BSA/PBS. The cells were then pretreated with PEG-catalase (200 units/mL), and treated with IFNγ (200 ng/mL) and LPS (1 µg/mL) at 37°C for the times indicated. Two micromolar DCFDA (Invitrogen) was added for the final 15 minutes of incubation. The reaction was halted by the addition of cold 1% BSA/PBS, cells were washed, and placed on ice until flow cytometric analysis.

### Glucose Oxidase (GOx) Assay

Immature DC were stimulated in complete medium without β-ME. Cells, 1×10^6^ cells/2 ml, were treated with 0.01 µg of GOx (Sigma-Aldrich) prior to IFNγ/LPS stimulation. Cell lysates were harvested at 15 and 45 minutes.

### Quantitative real-time PCR

RNA extraction (Qiagen, Valencia, CA) and 1-step quantitative real-time PCR (Applied Biosystems, Foster City, CA) were performed using manufactures protocols. Gene expression assays for amplifying mouse IL-12 were from Applied Biosystems. Real-time RT-PCR was done with the 7500 Real Time PCR System (Applied Biosystems). RPS29 was chosen to normalize relative quantification. Each sample was assayed in triplicate.

#### Statistical analysis

Means and standard error of the mean (SEM) for cell count and viability were determined. Differences between the group means were analyzed by the Student's t. (Prism 5, GraphPad Software, Inc. San Diego, CA).

## Supporting Information

Figure S1 OT-II T lymphocyte stimulation. OT-II CD4^+^ T lymphocytes and OVA_323–339_ -peptide-pulsed, IFNγ/LPS matured DC were co-cultured for 72 or 96 hours. T cell proliferation was determined by CFSE staining, the overall percentage of active cells was initially estimated by FSC v. SSC, the percentage of viable T cells was determined by positive staining for CD4 and PI exclusion (A) Representative flow cytometric analysis of 72 hour co-culture. (B) CFSE staining of proliferating OT-II lymphocytes (left −72 hours, right, 96 hours). (C) The levels of OT-II lymphocyte activation, viability and CD25/CD69 upregulation at 72 hours. The data are the mean (± SEM) percentage for 3 individual experiments with 3–4 of each genotype/experiment.
(PDF)

Figure S2 PCK inhibitor treatment. Supernatants from IFNγ/LPS-stimulated DC treated, or not, with Bisindolylmaleimide or PKCε inhibtor peptide were harvested from overnight cultures and assayed for secreted IL-12p70. The data are the mean (± SEM) for 3 individual experiments using pooled cells from 3–4 of each genotype/experiment with 2×10^5^ DC/0.2 ml.
(TIFF)
