# Supplementary figures and images for: NADPH Oxidase-2 Derived ROS Dictates Murine DC Cytokine-Mediated Cell Fate Decisions during CD4 T Helper-Cell Commitment
Source: PLoS One. 2011 Dec 1;6(12):e28198. doi: 10.1371/journal.pone.0028198 (PMC3228756; doi:10.1371/journal.pone.0028198)

A

WT

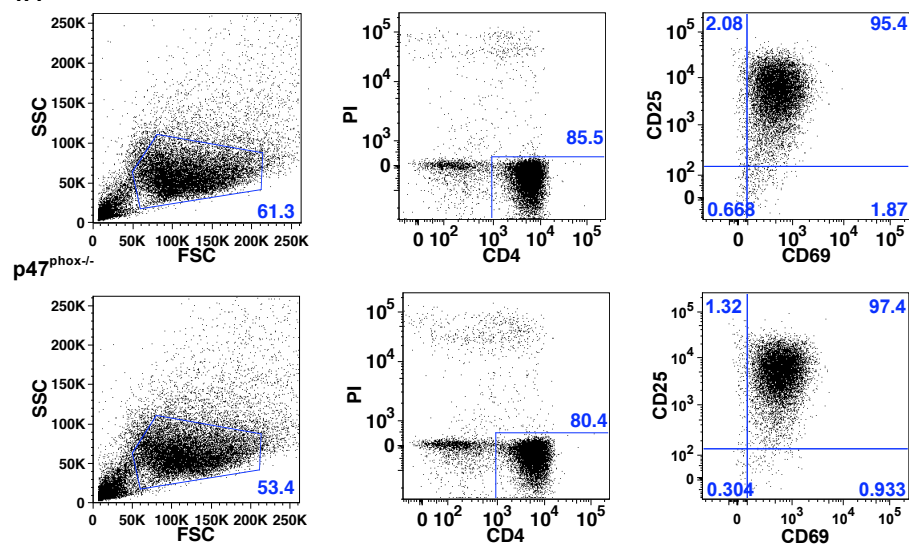

B

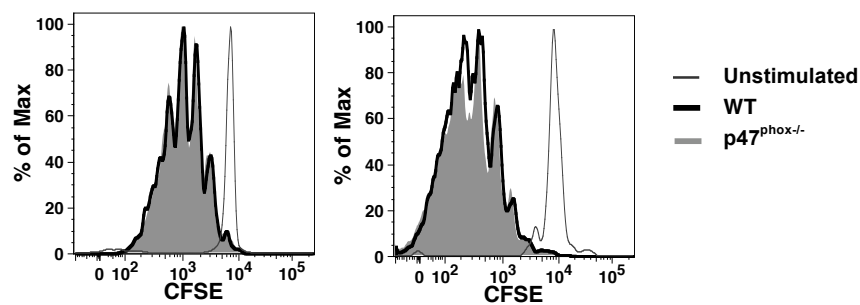

C

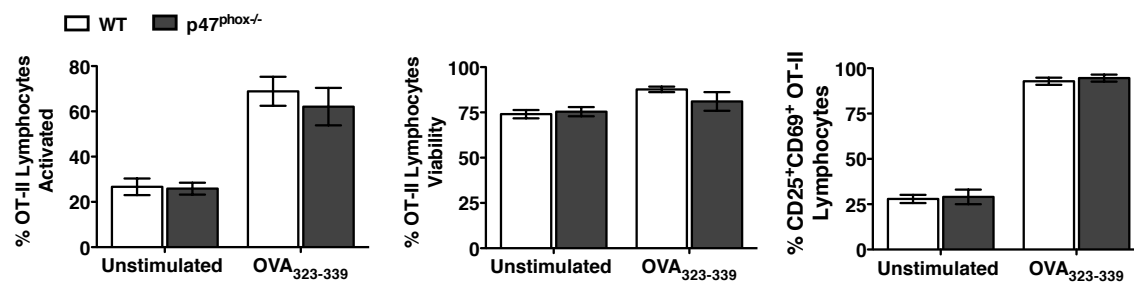

Supplement: Figure S1 — OT-II T lymphocyte stimulation. OT-II CD4+ T lymphocytes and OVA323–339 -peptide-pulsed, IFNγ/LPS matured DC were co-cultured for 72 or 96 hours. T cell proliferation was determined by CFSE staining, the overall percentage of active cells was initially estimated by FSC v. SSC, the percentage of viable T cells was determined by positive staining for CD4 and PI exclusion (A) Representative flow cytometric analysis of 72 hour co-culture. (B) CFSE staining of proliferating OT-II lymphocytes (left −72 hours, right, 96 hours). (C) The levels of OT-II lymphocyte activation, viability and CD25/CD69 upregulation at 72 hours. The data are the mean (± SEM) percentage for 3 individual experiments with 3–4 of each genotype/experiment. (PDF) [file pone.0028198.s001.pdf]

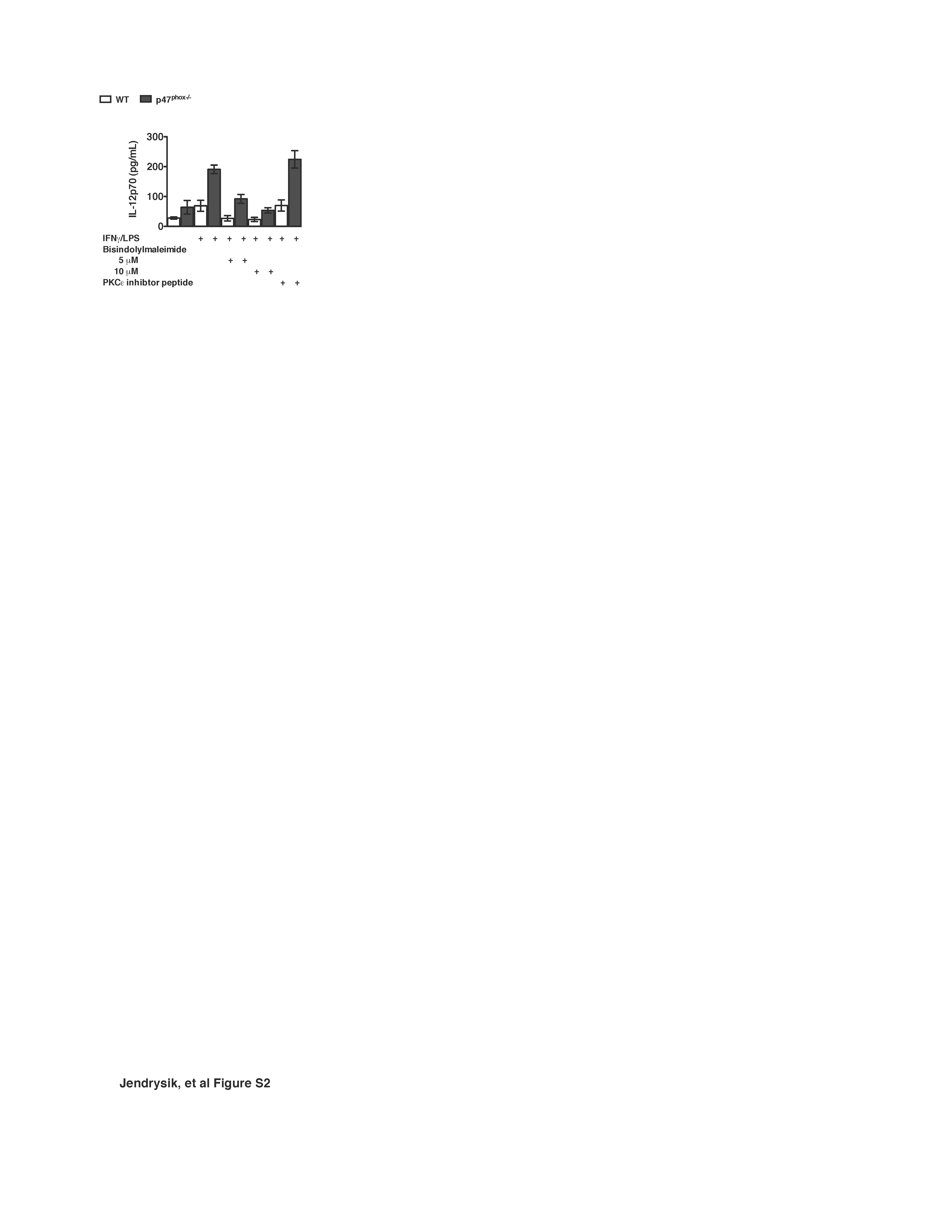

Supplement: Figure S2 — PCK inhibitor treatment. Supernatants from IFNγ/LPS-stimulated DC treated, or not, with Bisindolylmaleimide or PKCε inhibtor peptide were harvested from overnight cultures and assayed for secreted IL-12p70. The data are the mean (± SEM) for 3 individual experiments using pooled cells from 3–4 of each genotype/experiment with 2×10^5^ DC/0.2 ml. (TIFF) [file pone.0028198.s002.tiff]
